# Whole-Genome Sequence of Marine Bacterium *Phaeodactylibacter xiamenensis* Strain KD52, Isolated from the Phycosphere of Microalga *Phacodactylum tricornutum*

**DOI:** 10.1128/genomeA.01289-14

**Published:** 2014-12-11

**Authors:** Zhangran Chen, Xueqian Lei, Yi Li, Jingyan Zhang, Huajun Zhang, Luxi Yang, Wei Zheng, Hong Xu, Tianling Zheng

**Affiliations:** State Key Laboratory for Marine Environmental Sciences and Key Laboratory of the Ministry of Education for Coastal and Wetland Ecosystems, School of Life Sciences, Xiamen University, Xiamen, China

## Abstract

*Phaeodactylibacter xiamenensis* KD52 is a novel bacterium isolated from a culture of the alga *Phaeodactylum tricornutum* in Xiamen, Fujian Province, China. Here, we present the first draft genome sequence of this strain, which will provide an opportunity to further understand the functional genes related to signing for nutrition from the host algae and the molecular mechanisms underlying its beneficial properties.

## GENOME ANNOUNCEMENT

*Phaeodactylum tricornutum* has drawn more attention recently as a biofuel candidate due to several advantages, including less space required for growth, the use of nonarable land, salt water growth, ease of handling in liquid medium, high photosynthetic efﬁciency, and high growth rate (1, 2). Previous studies have reported that bacteria in the phycosphere play an important role in the growth of the microalgae (3, 4). Research involved in the relationship between microalgae and bacterium will facilitate better utilization of the beneficial materials of these organisms. *Phaeodactylibacter xiamenensis* KD52, a novel genus in the family *Saprospiraceae*, was isolated from a culture of the alga *P. tricornutum* and the strain was deposited in the KCTC/MCCC Bacteria Collection, with the accession number KCTC 32575 (5). Until now, there has been no reported genome sequence related to *Phaeodactylibacter xiamenensis*. To further understand the role of the bacterium in the phycosphere and its beneficial properties, the whole-genome sequence of strain KD52 was first determined in this study.

The draft genome of *Phaeodactylibacter xiamenensis* KD52 was sequenced with a whole-genome shotgun strategy using an Illumina HiSeq2000 instrument. *De novo* assembly was performed using the SOAPdenovo2 alignment tool (6). Based on the assembled result of strain KD52, we found that its genome size was 7,958,858 bp, with a 51.01% G+C content and 164 contigs. The results showed that the genome of strain KD52 contained 6,571 genes. The total length of genes was 7,067,088 bp, making up 88.80% of the genome. There were 378 tandem repeat sequences, with a total length of 132,087 bp, making up 1.6596% of genome. There were 159 minisatellite DNAs, 35 microsatellite DNAs, 45 tRNAs, and 9 rRNAs. All the assembled data were deposited in the NCBI nucleotide sequence database. Each gene was annotated through BlastP searches against the Swiss-Prot, COG, and KEGG databases. A total of 1,485 genes and 2,671 genes were assigned to COG classes and KEGG pathways, respectively.

Gene ontology (GO) searches were performed using all complete coding sequences. Results revealed that 44%, 19%, and 37% of genes were related to biological processes, cellular components, and molecular functions, respectively. From the GO category of biological processes, “cellular process” and “metabolic process” were the most dominant terms, representing 63% of genes. In the “cellular component” category, 90% of the genes were related to “cell” and “cell part.” Based on their molecular functions, 44% of the genes were identified as encoding proteins with catalytic activities and 40% of the genes were identified as encoding proteins with binding activity. A total of 112 genes were identified to be involved in macromolecular complex production, 244 genes were found for response to stimulus, and 157 genes were found for signing. The whole-genome sequence of the novel marine strain KD52 will promote understanding of genes involved in signing for nutrition from the host algae.

### Nucleotide sequence accession numbers.

This whole-genome shotgun project has been deposited at DDBJ/EMBL/GenBank under the accession number JPOS00000000. The first version (accession number JPOS01000000) is described in this paper. The strain of *Phaeodactylibacter xiamenensis* KD52 is available from the corresponding author (State Key Laboratory of Marine Environmental Science and Key Laboratory of the Ministry of Education for Coastal and Wetland Ecosystems, School of Life Sciences, Xiamen University, Xiamen, China).
